# Statin-Based Palliative Therapy for Hepatocellular Carcinoma

**DOI:** 10.1097/MD.0000000000001801

**Published:** 2015-10-23

**Authors:** Joni Yu-Hsuan Shao, Fei-Peng Lee, Chia-Lun Chang, Szu-Yuan Wu

**Affiliations:** From the Graduate Institute of Biomedical Informatics, Taipei Medical University, Taipei, Taiwan, R.O.C. (JY-HS); Institute of Toxicology, College of Medicine, National Taiwan University, Taipei, Taiwan, R.O.C. (S-YW); Department of Otorhinolaryngology, Wan Fang Hospital, Taipei Medical University, Taipei, Taiwan, R.O.C. (F-PL); Department of Hemato-Oncology, Wan Fang Hospital, Taipei Medical University, Taipei, Taiwan, R.O.C. (C-LC); Department of Radiation Oncology, Wan Fang Hospital, Taipei Medical University, Taipei, Taiwan, R.O.C. (S-YW); Department of Internal Medicine, School of Medicine, College of Medicine, Taipei Medical University, Taipei, Taiwan, R.O.C. (S-YW); Department of Biotechnology, Hungkuang University, Taichung, Taiwan, R.O.C. (S-YW); and Division of Gastroenterology, Department of Internal Medicine, Wan Fang Medical Center, Taipei Medical University, Taipei, Taiwan; (G-SL, F-MS, M-SW, T-SC, C-NC).

## Abstract

Most hepatocellular carcinoma (HCC) patients worldwide do not receive curative treatments. Alternative treatments for most HCC patients include palliative treatments, such as transarterial chemoembolization (TACE), chemotherapy, and radiotherapy. Although statins may be a chemopreventive treatment option for reducing hepatitis B virus (HBV)- and hepatitis C virus (HCV)-related HCC risks, their therapeutic effects are unknown. This study evaluated the effects of statin on HCC patients receiving palliative treatment.

Data from the National Health Insurance claims database and cancer registry databases of The Collaboration Center of Health Information Application, Taiwan, were analyzed. We included HCC patients who were treated between January 1, 2001, and December 31, 2010, and followed them from the index date to December 31, 2012. The inclusion criteria were presence of HBV carrier-related HCC, age >20 years, and having received TACE, radiotherapy, or chemotherapy as palliative treatment. The exclusion criteria were cancer diagnosis before HCC was confirmed, surgery, liver transplantation, radiofrequency ablation, or percutaneous ethanol injection as curative treatment, missing sex-related information, HCC diagnosis before HBV, and age <20 years. We enrolled 20,200 HCC patients.

The median follow-up duration was 1.66 years (interquartile range, 0.81). In total, 1988 and 18,212 patients received palliative treatment with and without statin use, respectively. HCC patients who received palliative treatment with statin use had lower HCC-specific deaths in all stages than those who received palliative treatment without statin use (*P* = 0.0001, 0.0002, 0.0012, and 0.0002, and relative risk (RR) = 0.763, 0.775, 0.839, and 0.718, for stages I–IV, respectively). In all-cause and HCC-specific deaths, decreasing trends (*P* for trend <0.0001) of adjusted hazard ratios (aHRs) were observed in all stages with no treatment, statin use only, palliative treatment only, and palliative treatment plus statin use. The aHRs of all-cause and HCC-specific deaths increased with the progress in cancer stage and reduced with the use of advanced therapeutic modalities (*P* for trend <0.0001). Differences in HBV- and non-HBV-related HCC were solely due to statin use. Statin use alone reduced HCC-specific deaths by 36% in non-HBV-related HCC in stage I and 50% in HBV-related HCC in stages II and III. With a relatively substantial reduction in mortality, the therapeutic effects of statin use were stronger in HBV-related HCC than in non-HBV-related HCC.

Palliative treatments are critical for HCC patients. Multiple therapeutic methods with statin use reduced the mortality risk. Statins prolong the survival of patients with advanced HCC receiving palliative treatment, thus demonstrating its therapeutic value as an adjuvant treatment. Furthermore, statin-based palliative treatment in early stage HCC remarkably reduced the number of deaths. For patients who cannot tolerate palliative treatments, statin use only might possibly reduce mortality, particularly in HBV-related early stage HCC patients (>50% reduction in HCC deaths).

## INTRODUCTION

In Taiwan, hepatocellular carcinoma (HCC) has been the second leading cause of death.^1^ The etiology of HCC in Taiwan differs from that in the West because HCC is increasingly being associated with hepatitis B virus (HBV) infections in Asia.^2^ Because HBV infections are endemic to Asia, more than 80% of HCC cases are encountered in this region.^2^ A considerable section of the population is at the risk of chronic liver disease and therefore HCC. Several Asia countries lack effective screening programs; therefore, the majority of HCC patients are diagnosed at an advanced stage, making treatment challenging. Palliative therapy is the first-line treatment for such patients. In the current era, numerous options are available for the palliative management of HCC. Potentially curative HCC treatments include surgical resection, liver transplantation, and local ablation; however, <20% of HCC cases receive these treatments, specifically those with early-stage disease, compensated liver disease, or few comorbidities.^3,4^ In addition to surgery, therapeutic modalities, such as percutaneous ethanol injection (PEI) and radiofrequency ablation (RFA) are potentially curative. *Curative* usually implies complete local control of the original lesion. Radiotherapy, chemotherapy, and liver transarterial chemoembolization (TACE) are *palliative* (ie, incomplete local control of the original lesion).^4^ However, most patients in Taiwan receive palliative treatment at presentation because of poor liver function, bilobular HCC, invasion of the major vessels, overt extrahepatic metastases, and underlying complicated comorbidities.

Statins have been reported to reduce HCC risks in HBV and HCV carriers.^5,6^ However, statin is widely adopted for its preventive effects and not for treatment or additive effects with other medical treatments. Numerous epidemiologic studies have reported the chemopreventive effects of statins on HCC risk, and basic studies have reported their anticancer effect on HCC cells and animal models.^2,5–9^ However, currently, no clinical or epidemiologic data clarifying the effect of statins and palliative treatments, such as palliative radiotherapy, chemotherapy, and TACE, are available. If HCC patients cannot receive curative treatments (eg, liver transplantation, RFA, PEI, and surgery), statin use might be a viable option. Whether statin use exerts an additive effect with other palliative treatments remains unclear. Furthermore, the group of patients who can benefit from statin use and the recommended dosage for effective treatment both warrant clarification. To address these topics, our study estimated the effect of statin use in HCC patients who received palliative treatment. Davila et al reported that globally, >70% of HCC patients do not receive curative treatments.^4^ Alternative treatments include palliative treatments, such as TACE, chemotherapy, and radiotherapy. Survival benefits can be achieved through palliative treatments, although a complete response and tumor ablation are impossible.^4,10,11^ Thus, identifying a safe, additive therapeutic drug exerting palliative effects is critical for HCC patients.

## PATIENTS AND METHODS

Two cohorts were formed from the combined data of the Taiwan National Health Insurance (NHI) database and cancer registry databases. These two databases cover approximately 99% of the entire population of Taiwan. We included HCC patients treated between January 1, 2001, and December 31, 2010, and followed them from the index date to December 31, 2012. The NHI Bureau releases research-oriented data sets, comprising all original claims data and registration files of the beneficiaries, through the Collaboration Center of Health Information Application (CCHIA). Taiwan launched the CCHIA program in 1995, and 99% of the population was covered by the end of 2012. The CCHIA thus allows researchers to trace medical service utilization by HCC patients in Taiwan. Abundant cancer-related information, such as the clinical stage, treatment modalities, pathological data, death from specific diseases, chemotherapy regimens, and concurrent or sequential chemotherapy or radiotherapy, is available in the cancer registry database. The enrolled patients were verified by the NHI and CCHIA, and the newly diagnosed HCC patients had no other cancers or distant metastasis. Data of patients or care providers are released to researchers after scrambling. Data are guarded by the encryption and individual identification is almost impossible. Agreement is necessary for all researchers who want to use NHI/CCHIA and demonstrate no intention to obtain information that could be potentially against the privacy of care providers or patients. The inclusion criteria were presence of HBV carrier-related HCC (International Classification of Diseases, Ninth Revision, Clinical Modification [ICD-9-CM] 070.2, 070.3, and V02.61 for hepatitis and 155.0 and 155.2; International Classification of Diseases for Oncology, Third Edition, C22.0 and C22.1 for cancer), age >20 years, and having received TACE, radiotherapy, or chemotherapy as palliative treatment. All HBV patients without a subsequent outpatient or emergency visit for a diagnosis of HBV within 12 months were excluded because they were considered to not have chronic hepatitis. In addition, non-HBV-related HCC cases were analyzed. The initial date of the first palliative treatment was considered the index date of HCC for HCC patients. The exclusion criteria were cancer diagnosis before HCC was confirmed, surgery, liver transplantation, RFA or PEI as curative treatment, missing sex-related information, HCC diagnosis before HBV, and age <20 years. We enrolled 20,200 HCC patients and categorized the HCC patients who received palliative treatment into statin and nonstatin users. The index date of statin use was the date of HCC confirmation. The aim of our study was to evaluate the effects of statin use in HCC patients who received palliative treatment such as TACE, chemotherapy, and radiotherapy. The primary endpoint was disease-free survival, and secondary endpoints were survival benefits if statins were used in HBV- or non-HBV-related HCC. The defined daily dose (DDD), recommended by the WHO, is a measure of the prescribed drug amount. DDD is the assumed average maintenance dose per day of a drug consumed for its main indication in adults.^12^ To examine the dose–response relationship, because the duration of the refill card was 3 months, we categorized statin and metformin use into 4 groups in each cohort (<28, 28–90, 91–365, and >365 cumulative [c]DDD). Patients who received <28 cDDD were defined as nonstatin users.^13^

Possible confounding factors were the following comorbidities: HBV (ICD-9-CM 0702 and 0703 and A-CODE V0261), HCV (ICD-9-CM 07041, 07044, 07051, and 07054, and A-CODE V0262 and 0707), other viral hepatitis (A-CODE V0269), alcohol-related diseases (ICD-9-CM 291, 303, 305, 5710, 5711, 5712, and 5713, and A-CODE A213, A215, and A347), acute coronary syndrome (ICD-9-CM 410-414), cerebrovascular accident (ICD-9-CM 430–438), chronic obstructive pulmonary disease (COPD, ICD-9-CM 490-496), diabetes mellitus (ICD-9-CM 250 and A-CODE A181), liver cirrhosis (ICD-9-CM 571.2, 571.5, 571.6, 572.2, 572.3, 572.4, 572.8, and 573.0), liver failure (ICD-9-CM 570), renal failure (ICD-9-CM 584–586), hypertension (ICD-9-CM 401–405), hyperlipidemia (ICD-9-CM 272.0–272.2), and peptic ulcers (ICD-9 531–534). Comorbidities before and after 6 months of the HCC index date, classified according to the main diagnosis code on the first admission or >2 repeated diagnoses in the outpatient department, were included. In addition, the analyses were stratified by and adjusted for age, sex, American Joint Committee on Cancer (AJCC) clinical cancer stage, comorbidities, and the use of metformin, aspirin, acetylcholinesterase (ACE) inhibitors, and anti-HBV or HCV drugs.

The cumulative incidence of death was estimated using the Kaplan–Meier method, and statin use and nonuse were compared using a log-rank test. Cox proportional hazard regression was used for calculating the hazard ratios (HRs) of death among HBV-related HCC patients receiving palliative treatment with and without statin use. In the multivariate analysis, the HRs were adjusted for age, sex, baseline comorbidities, clinical stage, and the use of metformin, aspirin, ACE inhibitors, anti-HBV, or anti-HCV drugs. The effect of statin use was stratified and analyzed for HBV- and non-HBV-related HCC patients of similar age and clinical stage. All analyses were performed using SAS version 9.3 (SAS, Cary, NC). Two-tailed *P* < 0.05 was considered statistically significant.

## RESULTS

The median follow-up duration was 1.66 years (interquartile range, 0.81). Furthermore, 1988 and 18,212 patients received palliative treatment with and without statin use, respectively (Table 1). Mean patient age, follow-up duration, aspirin and metformin use, and the presence of severe comorbidities (eg, liver cirrhosis, hypertension, diabetes, COPD, acute coronary syndrome, and cerebral vascular disease) were all higher in the statin group than in the nonstatin group. HCC deaths (disease-specific survival) were significantly higher in the nonstatin group than in the statin group.

**TABLE 1:**
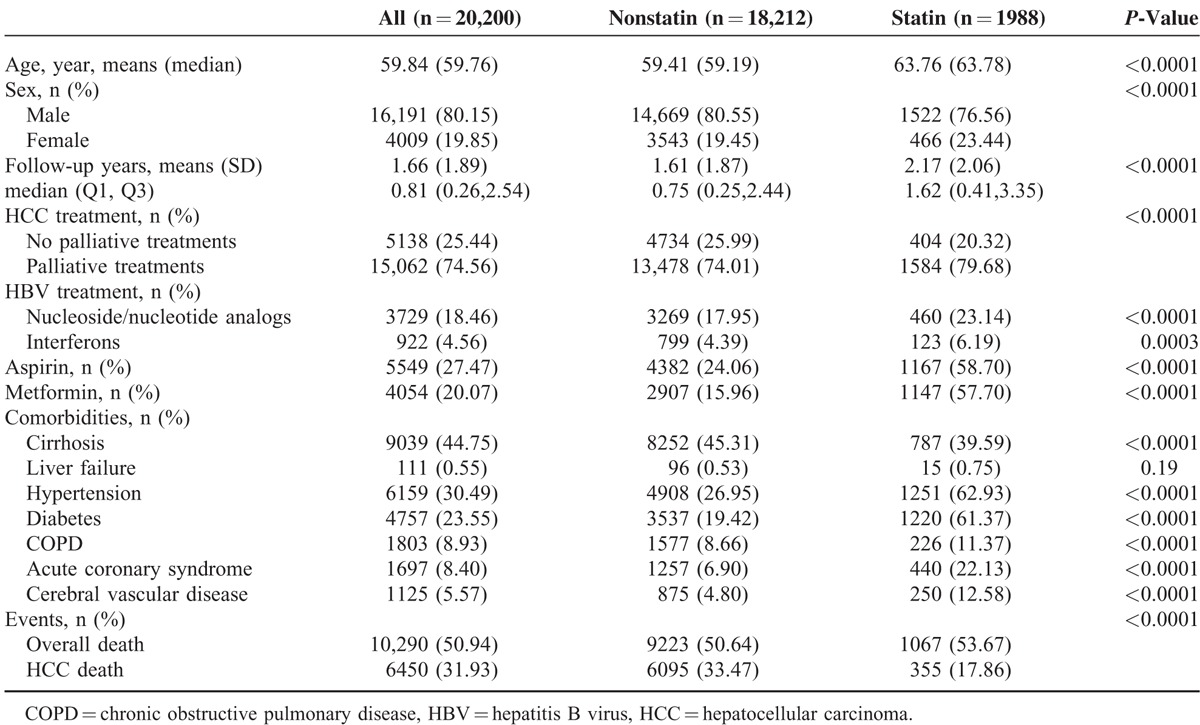
Baseline Characteristics and Outcomes of Study Cohort by Statin Status

Furthermore, we estimated the risks of HCC-specific and all-cause deaths according to both statin use status and HCC treatment stratified by the AJCC stage (Table 2). HCC patients who received palliative treatment with statin use had a significant overall survival benefit in all stages compared with those who received palliative treatment without statin use. The relative risks (RRs) of all-cause death reduced in all stages in HCC patients who received palliative treatment with statin use compared with those who received palliative treatment without statin use (*P* = 0.0001, 0.0002, 0.0012, and 0.0002, and RR = 0.763, 0.775, 0.839, and 0.718, respectively, for stages I–IV). Among patients who received no palliative treatment, statin use alone did not reduce the risk of all-cause death in stages I, II, and IV. HCC-specific deaths among HCC patients who received palliative treatment with statin use were lower in all stages compared with those who received palliative treatment without statin use (*P* < 0.0001, <0.0001, <0.0001, and =0.0054, and RR = 0.409, 0.445, 0.632, and 0.649, respectively, for stages I–IV; Table 2). Among HCC patients who did not receive palliative treatment but received statin, HCC-specific deaths were not significantly low in advanced HCC stages III and IV. However, statin use alone in the early stages resulted in significant reductions in HCC-specific deaths compared with no palliative treatment and no statin use (*P* = 0.014 and 0.016, and RR = 0.465 and 0.418, respectively, for stages I and II).

**TABLE 2:**
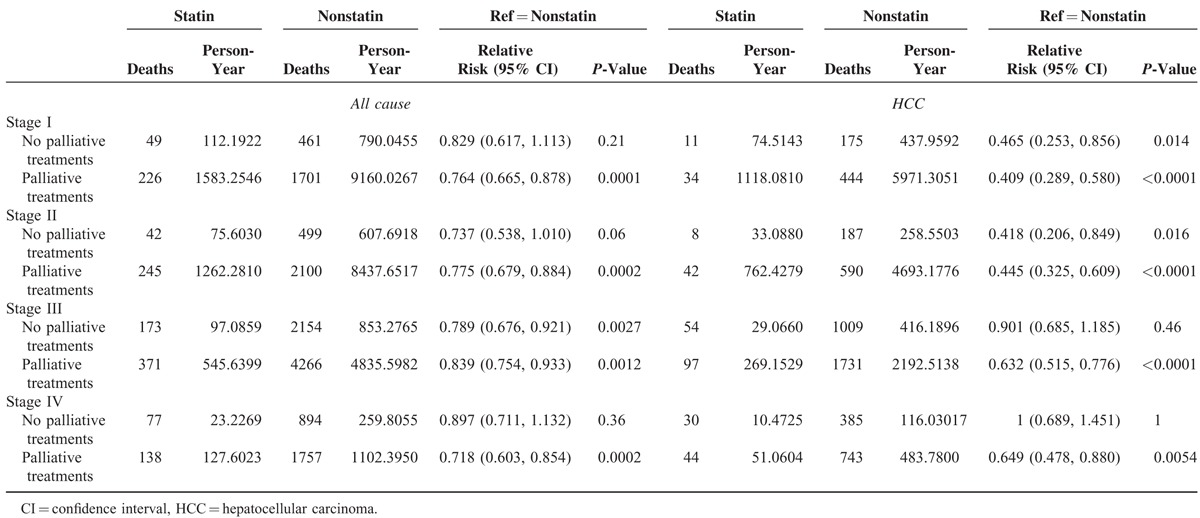
Risk of HCC-Related and All-Cause Deaths by Statin Status and HCC Treatment

To understand the effect of statin use in HBV- and non-HBV-related HCC patients receiving palliative treatment, the HCC groups were classified into HBV- and non-HBV-related HCC cases (Tables 3 and 4, respectively). All-cause death in HBV-related HCC were analyzed, and our data demonstrated a significant decreasing trend (*P* for trend <0.001) for aHRs in all stages for no palliative treatment, statin only, palliative treatment, and palliative treatment plus statin use (Table 3). The aHRs of all-cause death were reduced in all stages in HCC patients who received palliative treatment with statin use compared with those who received no treatment (*P* < 0.0001, <0.0001, =0.0001, and <0.0001 and aHR = 0.281, 0.274, 0.445, and 0.428, respectively, for stages I–IV). The aHRs of all-cause death increased with cancer stage and reduced with the use of advanced therapeutic modalities (*P* for trend <0.0001). No significant differences were observed between patients with statin use and patients with no treatment among stage IV HBV-related HCC patients. For HCC-specific deaths, a decreasing trend (*P* for trend <0.001) was observed in all stages with no treatment, statin use alone, palliative treatment, and palliative treatment plus statin use. The aHRs of HCC deaths were lower in all stages in HCC patients who received palliative treatment with statin use compared with those who received no treatment (*P* < 0.0001, <0.0001, =0.0001, and <0.0001, and aHR = 0.116, 0.132, 0.385, and 0.403, respectively, for stages I–IV). The aHRs of HCC deaths increased with the cancer stage and reduced with the use of advanced therapeutic modalities (*P* for trend <0.0001). Statin use alone in the early stages significantly reduced the number of HCC deaths compared with no treatment (*P* = 0.015 and 0.008, and aHR = 0.464 and 0.381, respectively, for stages I and II), and statin use alone significantly reduced HCC deaths in advanced stages III and IV in HBV-related HCC patients compared with no treatment. The outcomes in the non-HBV-related HCC patients were similar to those in the HBV-related HCC patients (Table 4). Multiple therapeutic methods resulted in the lowest risks for all-cause and HCC-specific deaths. Advantages of multiple therapeutic methods were distinct in the early stages. The aHRs of all-cause death reduced in all stages in HCC patients who received palliative treatment with statin compared with those who received no treatment (*P* < 0.0001, <0.0001, =0.0001, and <0.0001, and aHR = 0.285, 0.347, 0.476, and 0.489, respectively, for stages I–IV). The aHRs of all-cause death increased with the cancer stage and reduced with the use of advanced therapeutic modalities (*P* for trend <0.0001). The aHRs of HCC-specific deaths were lower in all stages in HCC patients who received palliative treatment with statin use compared with those who received no treatment (*P* < 0.0001, <0.0001, =0.0001, and <0.0001, and aHR = 0.144, 0.210, 0.364, and 0.420, respectively, for stages I–IV). The aHRs of HCC deaths increased with the cancer stage and reduced with the use of advanced therapeutic modalities (*P* for trend <0.001). Differences in HBV-related HCC and non-HBV-related HCC deaths were only observed with statin use. In non-HBV-related HCC deaths, even in early stage II HCC, statin use alone did not significantly reduce HCC deaths. Statin use alone in non-HBV-related HCC cases reduced HCC deaths by 36% in stage I (Table 4). For HBV-related HCC patients in stages I and II, statin use alone reduced HCC deaths by >50% (Table 3).

**TABLE 3:**
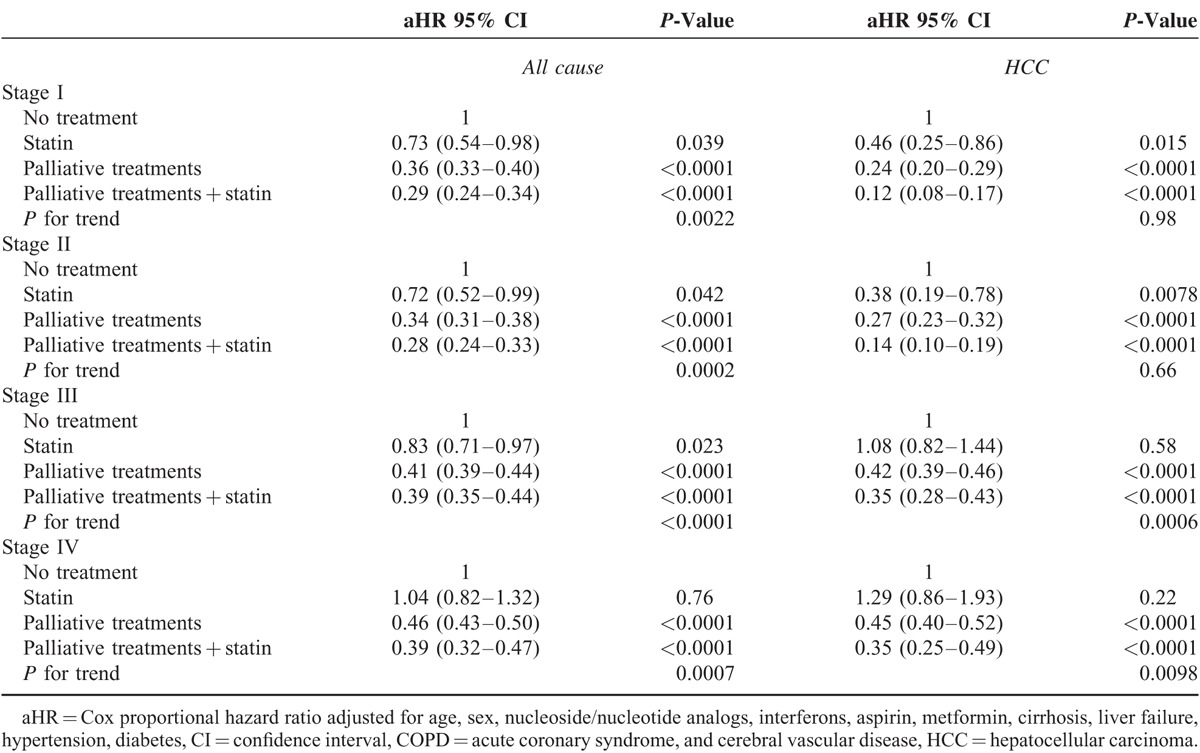
Risk of HCC Death and All-Cause Deaths by Statin Status and HBV-Related HCC Treatment

**TABLE 4:**
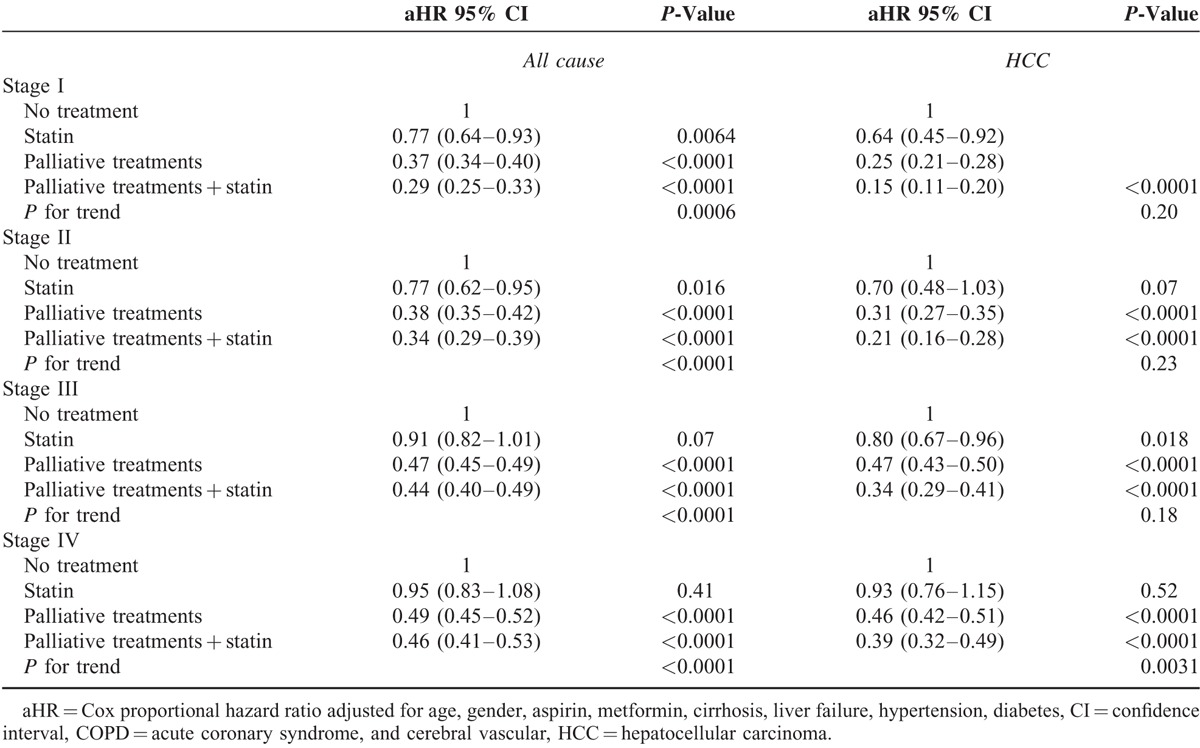
Risk of HCC Death and All-Cause Deaths by Statin Status and HBV-Related HCC Treatment

**FIGURE 1:**
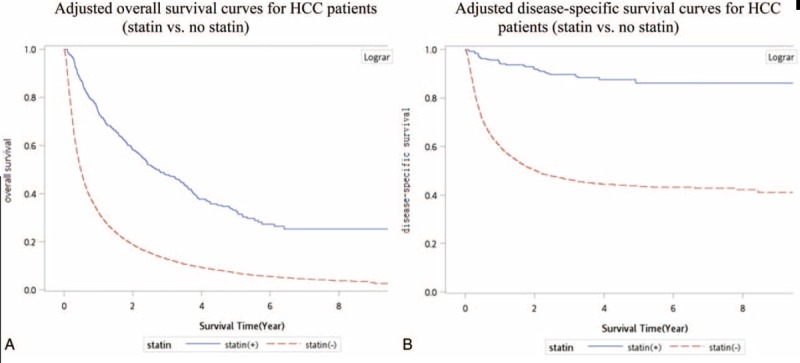
(A) Adjusted overall survival curves for hepatocellular carcinoma patients (statin vs. no statin use). (B) Adjusted disease-specific survival curves for hepatocellular carcinoma patients (statin vs. no statin use).

In HCC patients who received palliative treatment with statin use, the palliative treatments may have contributed to high overall and disease-specific survival rates (Fig. 1A and B). The 5-year overall rate increased from 9% to 38% (log-rank test *P* < 0.0001) in HCC patients who received palliative treatment with statin use compared with those who received palliative treatment without statin use. The 5-year disease-specific survival rate increased from 44% to 83% (log-rank test *P* < 0.001) in HCC patients who received palliative treatment with statin use.

## DISCUSSION

Curative treatments in Taiwan are received only by a minority of HCC patients; the majority receive palliative treatments,^3,4^ the survival benefits of which have been reported by numerous studies.^14–16^ Because these medical modalities (TACE, radiotherapy, and chemotherapy) are palliative, the true survival benefits remain unclear. HCC has a high incidence of local recurrence, necessitating complex, and multiple treatments for HCC patients. Most HCC patients repeatedly receive different palliative treatments; single palliative treatments for HCC patients are rare. For example, the efficacy of TACE is limited by vascular shunting, recanalization around the tumor capsule, and the development of multiple feeding vessels. TACE is repeated to overcome these limitations.^17^ However, it frequently results in the outgrowth of HCC refractory to TACE. In such a state, chemotherapy or radiotherapy may be necessary.^11,17^ Meng et al^11^ reported that TACE combined with radiotherapy was therapeutically more beneficial and that it significantly improved the survival and tumor responses of patients than TACE alone. Hence, we grouped all palliative treatments into a single group called *palliative treatment*. Identifying a safe and additive therapeutic drug for these palliative treatments would help HCC patients achieve superior survival and tumor control. Statins might be a chemopreventive regimen for reducing HBV- and HCV-related HCC risks, but their therapeutic effects are unknown. According to a review of relevant literature, this is the first article on the therapeutic effects of statin use alone and palliative treatment with and without statin use.

The mechanism through which statin use decreases the HCC risk in patients is unknown; possible mechanisms are inhibiting downstream products of the mevalonate pathway,^18–21^ triggering tumor-specific apoptosis,^22^ inhibiting the proteasome pathway,^23^ and inhibiting cholesterol synthesis and HBV and HCV replication.^24,25^ On the basis of these basic theories, clinical and epidemiologic data were generated.^9,13,26^ Many studies have demonstrated the chemopreventive effect of statins in lowering both HBV- and HCV-related HCC risk.^2,5,6,27^ However, few studies have reported the therapeutic effect of statin use in HCC patients. The effect of statin use alone for HCC treatment is not clearly understood. In our study, statin use alone for early stage HCC exerted a therapeutic effect (Table 2). The therapeutic effect of statin use alone might have been more distinct in HBV-related HCC patients than in HCV-related HCC patients (Table 3). HCC deaths reduced by >50% in HBV-related HCC patients (Table 3), and in non-HBV-related HCC, statin use alone resulted in a >30% reduction in HCC deaths (Table 4), implying that among HCC patients with multiple underlying diseases who cannot tolerate TACE, chemotherapy, or radiotherapy, statin use solely might be helpful in the early stages. Statin use in HBV-related HCC had a superior therapeutic effect. For advanced-stage HCC patients, statin use alone was insufficient to reduce HCC deaths; other palliative treatments should be considered for reducing such HCC deaths. To our knowledge, this is the first article on the advantages of statin alone for HBV-related early-stage HCC patients.

Few studies have examined the combination of statin use with palliative treatment, such as radiotherapy, chemotherapy, and TACE. Only one randomized controlled trial reported that statin use with TACE or systemic chemotherapy significantly contributed to survival.^28^ Statin use with TACE might prolong the survival of patients with advanced HCC. These results are compatible with those of the present study (Tables 2–4); the only difference was that our palliative treatment included radiotherapy. With radiotherapy, statin use enhances radiation, inhibits cancer cell motility, and improves survival, as has been demonstrated in prostate and rectal cancers and glioblastoma multiforme.^29–36^ Data on the additive effect of statin in HCC patients who receive radiotherapy is lacking. Although our outcomes cannot denote the radiation-enhancing effect on HCC, our indirect evidence reveals that statin use and radiotherapy might exert an additive effect against HCC.

In Table 1, the presence of comorbidities was higher in the statin group than in the other groups. Theoretically, the statin group might have a shorter survival time compared with the nonstatin group because of the high number of underlying diseases. Even with the presence of severe comorbidities, such as liver cirrhosis, hypertension, diabetes, COPD, acute coronary syndrome, and cerebral vascular disease, the overall and disease-specific survival rates were higher in the statin group than in the nonstatin group. Thus, the therapeutic effects of statin use were underestimated in our analysis; statin use should be more effective in individuals with HCC lacking comorbidities. Randomized clinical trials in HCC populations who receive palliative treatment with statin use are thus warranted.

Palliative therapeutic modalities with statin use were more effective for reducing HCC deaths compared with palliative treatment without statin use (Table 2), particularly in the advanced stages (stages III and IV). Association of palliative treatment with reduced mortality in our study was similar to the results of Davila's study.^4^ In HCC patients who received only statin, HCC-specific deaths did not reduce significantly in HCC stages III and IV. Thus, statin alone is insufficient, rendering patient selection for sole statin use critical. No significant differences were observed in the different treatment modality groups, irrespective of whether they received no treatment, statin use only, palliative treatment, or palliative treatment with statin use, when stratified by clinical state (Table 3). Our data demonstrated a significant decreasing trend (*P* for trend <0.0001) of aHRs in all stages with no treatment, statin use only, palliative treatment, and palliative treatment plus statin use. Multiple therapeutic methods resulted in low risks of all-cause or HCC-specific deaths. Advantages of multiple therapeutic methods were distinct in the earlier stages. The aHRs of all-cause and HCC-specific deaths increased with the cancer stage and reduce with the use of advanced therapeutic modalities (*P* for trend <0.001; Tables 3 and 4). Differences in HBV- and non-HBV-related HCC were observed with statin use alone. In non-HBV-related HCC, including early stage II HCC, sole statin use did not significantly reduce HCC deaths. Statin use alone in non-HBV-related HCC reduced HCC deaths by 36% in stage I (Table 4). For HBV-related HCC patients, statin use alone in stages I and II reduced HCC deaths by 50% (Table 3). Yielding a highly reduced mortality, the therapeutic effect of statin use was stronger in HBV-related HCC patients. Overall survival with statin use combined with palliative treatment remained low after 5 years, a result attributable to old age and severe comorbidities in the statin group (Fig. 1A). For disease-specific survival, the survival curve plateaued after 5 years (Fig. 1B). Thus, statin use with palliative treatment might be a curative treatment in selected patients.

Use of multiple therapeutic methods resulted in the lowest mortality risk. The aHRs of all-cause and HCC-specific deaths increased with the cancer stage and reduced with the use of advanced modalities (*P* for trend <0.0001). Palliative treatments with statin use are valuable for inoperable and unresectable HCC patients, and their use highly reduced the mortality risk in the early cancer stages. If HCC patients are extremely weak and unable to tolerate radiotherapy, TACE, or chemotherapy, statin use alone in the early stages (stages I and II) can exert a therapeutic effect, particularly in HBV-related HCC patients (>50% reduction in HCC deaths). Statin use contributed to overall and disease-specific survival effects with palliative treatment (Fig. 1A and B) and prolonged the survival of patients with advanced HCC, which is indicative of its efficacy as an adjuvant treatment. The 5-year overall rate increased from 9% to 38% (log-rank test *P* < 0.0001) in HCC patients who received palliative chemotherapy, radiotherapy, or TACE with statin use compared with those who did not receive statin. The 5-year specific survival rate increased from 44% to 83% (log-rank test *P* < 0.0001) in HCC patients who received palliative treatment and statin use.

A limitation of this study is that the actual statin dose and duration were not measured; therefore, the actual dose–response effect was unclear. In addition, statins were not analyzed by type; thus, the potential effects of specific statins remain unknown. A large randomized trial with a suitable regimen in well-selected patients for comparing standard approaches is required to obtain such information. Moreover, the diagnoses of HCC and all other comorbidities were completely dependent on the ICD codes. Nevertheless, the enrolled patients were all confirmed by the NHI and CCHIA, and the NHI Bureau and CCHIA randomly review charts and interview patients to validate the diagnoses. Hospitals with outlier diagnoses and practices may be audited and subsequently be heavily penalized if malpractice and discrepancies are discovered. Another limitation is that the databases contain no information on tobacco use, alcohol consumption, dietary habits, socioeconomic status, educational level, and the body mass index, all of which may be risk factors in the survival analysis. However, given the magnitude and significance of the observed effects, these limitations are unlikely to have compromised the results.

In summary, palliative treatments are critical for HCC patients, and multiple therapeutic methods combined with statin use yielded the lowest mortality risk. Statin use prolonged the survival of patients with advanced HCC who received palliative treatment, which is indicative of its value as an adjuvant treatment. Palliative treatment with statin use in early-stage HCC presented the highest reduction in deaths and is more effective than other treatment options. For patients who cannot tolerate palliative treatments, statin use alone might be a possible mortality-reducing drug, particularly in HBV-related early-stage HCC patients (>50% reduction in HCC deaths).

## References

[1] Health Promotion Administration; Ministry of Health and Welfare. Taiwan Cancer Registry report. 2011 ed. Research funding for this work was done by Taipei Medical University-Wan Fang Hospital; 2011; http://www.hpa.gov.tw/BHPNet/Web/Stat/StatisticsShow.aspx?No=201404160001.

[2] Singh S Singh PP Singh AG Statins are associated with a reduced risk of hepatocellular cancer: a systematic review and meta-analysis. *Gastroenterology* 2013; 144:323–332. 23063971 10.1053/j.gastro.2012.10.005

[3] Liu JH Chen PW Asch SM Surgery for hepatocellular carcinoma: does it improve survival? *Ann Surg Oncol* 2004; 11:298–303. 14993025 10.1245/aso.2004.03.042

[4] Davila JA Duan Z McGlynn KA Utilization and outcomes of palliative therapy for hepatocellular carcinoma: a population-based study in the United States. *J Clin Gastroenterol* 2012; 46:71–77. 22157221 10.1097/MCG.0b013e318224d669 PMC3832893

[5] Tsan YT Lee CH Ho WC Statins and the risk of hepatocellular carcinoma in patients with hepatitis C virus infection. *J Clin Oncol* 2013; 31:1514–1521. 23509319 10.1200/JCO.2012.44.6831

[6] Tsan YT Lee CH Wang JD Statins and the risk of hepatocellular carcinoma in patients with hepatitis B virus infection. *J Clin Oncol* 2012; 30:623–630. 22271485 10.1200/JCO.2011.36.0917

[7] Cao Z Fan-Minogue H Bellovin DI MYC phosphorylation, activation, and tumorigenic potential in hepatocellular carcinoma are regulated by HMG-CoA reductase. *Cancer Res* 2011; 71:2286–2297. 21262914 10.1158/0008-5472.CAN-10-3367 PMC3059327

[8] Lonardo A Loria P Potential for statins in the chemoprevention and management of hepatocellular carcinoma. *J Gastroenterol Hepatol* 2012; 27:1654–1664. 22849701 10.1111/j.1440-1746.2012.07232.x

[9] Singh S Singh PP Roberts LR Chemopreventive strategies in hepatocellular carcinoma. *Nat Rev Gastroenterol Hepatol* 2014; 11:45–54. 23938452 10.1038/nrgastro.2013.143 PMC4334449

[10] Seong J Park HC Han KH Local radiotherapy for unresectable hepatocellular carcinoma patients who failed with transcatheter arterial chemoembolization. *Int J Radiat Oncol Biol Phys* 2000; 47:1331–1335. 10889387 10.1016/s0360-3016(00)00519-8

[11] Meng MB Cui YL Lu Y Transcatheter arterial chemoembolization in combination with radiotherapy for unresectable hepatocellular carcinoma: a systematic review and meta-analysis. *Radiother Oncol* 2009; 92:184–194. 19042048 10.1016/j.radonc.2008.11.002

[12] Darwish Murad S Kim WR Harnois DM Efficacy of neoadjuvant chemoradiation, followed by liver transplantation, for perihilar cholangiocarcinoma at 12 US centers. *Gastroenterology* 2012; 143:88.e3–98.e3.quiz e14. 22504095 10.1053/j.gastro.2012.04.008 PMC3846443

[13] Singh S Singh PP Statins for prevention of hepatocellular cancer: one step closer? *Hepatology* 2014; 59:724–726. 23839991 10.1002/hep.26614

[14] Nagano H Miyamoto A Wada H Interferon-alpha and 5-fluorouracil combination therapy after palliative hepatic resection in patients with advanced hepatocellular carcinoma, portal venous tumor thrombus in the major trunk, and multiple nodules. *Cancer* 2007; 110:2493–2501. 17941012 10.1002/cncr.23033

[15] Qin S Bai Y Lim HY Randomized, multicenter, open-label study of oxaliplatin plus fluorouracil/leucovorin versus doxorubicin as palliative chemotherapy in patients with advanced hepatocellular carcinoma from Asia. *J Clin Oncol* 2013; 31:3501–3508. 23980077 10.1200/JCO.2012.44.5643

[16] Toya R Murakami R Baba Y Conformal radiation therapy for portal vein tumor thrombosis of hepatocellular carcinoma. *Radiother Oncol* 2007; 84:266–271. 17716760 10.1016/j.radonc.2007.07.005

[17] Liao M Huang J Zhang T Transarterial chemoembolization in combination with local therapies for hepatocellular carcinoma: a meta-analysis. *PLoS One* 2013; 8:e68453. 23844203 10.1371/journal.pone.0068453 PMC3701086

[18] Chan KK Oza AM Siu LL The statins as anticancer agents. *Clin Cancer Res* 2003; 9:10–19. 12538446

[19] Danesh FR Sadeghi MM Amro N 3-Hydroxy-3-methylglutaryl CoA reductase inhibitors prevent high glucose-induced proliferation of mesangial cells via modulation of Rho GTPase/p21 signaling pathway: implications for diabetic nephropathy. *Proc Natl Acad Sci U S A* 2002; 99:8301–8305. 12048257 10.1073/pnas.122228799 PMC123062

[20] Blanco-Colio LM Munoz-Garcia B Martin-Ventura JL 3-Hydroxy-3-methylglutaryl coenzyme A reductase inhibitors decrease Fas ligand expression and cytotoxicity in activated human T lymphocytes. *Circulation* 2003; 108:1506–1513. 12952848 10.1161/01.CIR.0000089086.48617.2B

[21] Takemoto M Liao JK Pleiotropic effects of 3-hydroxy-3-methylglutaryl coenzyme a reductase inhibitors. *Arterioscler Thromb Vasc Biol* 2001; 21:1712–1719. 11701455 10.1161/hq1101.098486

[22] Wong WW Dimitroulakos J Minden MD HMG-CoA reductase inhibitors and the malignant cell: the statin family of drugs as triggers of tumor-specific apoptosis. *Leukemia* 2002; 16:508–519. 11960327 10.1038/sj.leu.2402476

[23] Rao S Porter DC Chen X Lovastatin-mediated G1 arrest is through inhibition of the proteasome, independent of hydroxymethyl glutaryl-CoA reductase. *Proc Natl Acad Sci U S A* 1999; 96:7797–7802. 10393901 10.1073/pnas.96.14.7797 PMC22141

[24] Ikeda M Abe K Yamada M Different anti-HCV profiles of statins and their potential for combination therapy with interferon. *Hepatology* 2006; 44:117–125. 16799963 10.1002/hep.21232

[25] Bader T Korba B Simvastatin potentiates the anti-hepatitis B virus activity of FDA-approved nucleoside analogue inhibitors in vitro. *Antiviral Res* 2010; 86:241–245. 20211652 10.1016/j.antiviral.2010.02.325 PMC2869246

[26] Carrat F Statin and aspirin for prevention of hepatocellular carcinoma: what are the levels of evidence? *Clin Res Hepatol Gastroenterol* 2014; 38:9–11. 24183917 10.1016/j.clinre.2013.09.007

[27] Chen CI Kuan CF Fang YA Cancer risk in HBV patients with statin and metformin use: a population-based cohort study. *Medicine (Baltimore)* 2015; 94:e462. 25674734 10.1097/MD.0000000000000462 PMC4602747

[28] Kawata S Yamasaki E Nagase T Effect of pravastatin on survival in patients with advanced hepatocellular carcinoma. A randomized controlled trial. *Br J Cancer* 2001; 84:886–891. 11286466 10.1054/bjoc.2000.1716 PMC2363838

[29] Mace AG Gantt GA Skacel M Statin therapy is associated with improved pathologic response to neoadjuvant chemoradiation in rectal cancer. *Dis Colon Rectum* 2013; 56:1217–1227. 24104995 10.1097/DCR.0b013e3182a4b236

[30] Larner J Jane J Laws E A phase I-II trial of lovastatin for anaplastic astrocytoma and glioblastoma multiforme. *Am J Clin Oncol* 1998; 21:579–583. 9856659 10.1097/00000421-199812000-00010

[31] Kollmeier MA Katz MS Mak K Improved biochemical outcomes with statin use in patients with high-risk localized prostate cancer treated with radiotherapy. *Int J Radiat Oncol Biol Phys* 2011; 79:713–718. 20452139 10.1016/j.ijrobp.2009.12.006

[32] Katz MS Minsky BD Saltz LB Association of statin use with a pathologic complete response to neoadjuvant chemoradiation for rectal cancer. *Int J Radiat Oncol Biol Phys* 2005; 62:1363–1370. 16029794 10.1016/j.ijrobp.2004.12.033

[33] Hamalukic M Huelsenbeck J Schad A Rac1-regulated endothelial radiation response stimulates extravasation and metastasis that can be blocked by HMG-CoA reductase inhibitors. *PLoS One* 2011; 6:e26413. 22039482 10.1371/journal.pone.0026413 PMC3198428

[34] Gutt R Tonlaar N Kunnavakkam R Statin use and risk of prostate cancer recurrence in men treated with radiation therapy. *J Clin Oncol* 2010; 28:2653–2659. 20421534 10.1200/JCO.2009.27.3003

[35] D’Amico AV Statin use and the risk of prostate-specific antigen recurrence after radiation therapy with or without hormone therapy for prostate cancer. *J Clin Oncol* 2010; 28:2651–2652. 20421530 10.1200/JCO.2010.28.5809

[36] Alizadeh M Sylvestre MP Zilli T Effect of statins and anticoagulants on prostate cancer aggressiveness. *Int J Radiat Oncol Biol Phys* 2012; 83:1149–1153. 22270166 10.1016/j.ijrobp.2011.09.042

